# Integrated Transcriptomics and Metabolomics Analysis of Two Maize Hybrids (ZD309 and XY335) under Heat Stress at the Flowering Stage

**DOI:** 10.3390/genes15020189

**Published:** 2024-01-30

**Authors:** Pu Zhao, Lei Sun, Siqi Zhang, Bo Jiao, Jiao Wang, Chunhong Ma

**Affiliations:** 1 Institute of Biotechnology and Food Science, Hebei Academy of Agriculture and Forestry Science/Hebei Key Laboratory of Plant Genetic Engineering, Shijiazhuang 050051, China; zhaopu2009@126.com (P.Z.); rayforlogin@163.com (L.S.); zhangsiqi202210@163.com (S.Z.); jiaobo1206@163.com (B.J.); xiangxiemaoshe@163.com (J.W.); 2 College of Agronomy and Biotechnology, Hebei Normal University of Science and Technology, Qinhuangdao 066000, China

**Keywords:** maize, heat stress, flowering stage, transcriptome, metabolomics

## Abstract

High temperature around flowering has a serious impact on the growth and development of maize. However, few maize genes related to flowering under heat stress have been confirmed, and the regulatory mechanism is unclear. To reveal the molecular mechanism of heat tolerance in maize, two maize hybrids, ZD309 and XY335, with different heat resistance, were selected to perform transcriptome and metabolomics analysis at the flowering stage under heat stress. In ZD309, 314 up-regulated and 463 down-regulated differentially expressed genes (DEGs) were detected, while 168 up-regulated and 119 down-regulated DEGs were identified in XY335. By comparing the differential gene expression patterns of ZD309 and XY335, we found the “frontloaded” genes which were less up-regulated in heat-tolerant maize during high temperature stress. They included heat tolerance genes, which may react faster at the protein level to provide resilience to instantaneous heat stress. A total of 1062 metabolites were identified via metabolomics analysis. Lipids, saccharides, and flavonoids were found to be differentially expressed under heat stress, indicating these metabolites’ response to high temperature. Our study will contribute to the identification of heat tolerance genes in maize, therefore contributing to the breeding of heat-tolerant maize varieties.

## 1. Introduction

High temperature is one of the most important factors affecting crop production worldwide under global warming [1]. Climate change increases the frequency and duration of high temperature stress, and it impacts crop yield negatively [2]. Maize (*Zea mays* L.) is a crucial cereal crop for food, feed, fuel, and industrial raw materials in the world, but it is sensitive to heat stress. Previous studies indicated that a 1 °C increase in temperature will lead to 7.4% yield loss in maize [3]. Heat stress has adverse effects on maize growth and development. Severe heat stress affects physiological processes such as seed germination, leaf elongation, reproductive development, ASI, photosynthesis, and grain-filling duration, ultimately leading to a decrease in yield and grain quality. At the cellular level, the rapid accumulation of reactive oxygen species (ROS) leads to cellular membrane damage, protein misfolding and degradation, and the inhibition of chlorophyll synthesis [4].

Recent studies have described the key stages of maize and explain the yield loss caused by heat stress. Both short-term and long-term heat stress have a negative impact on the reproductive process and harvest index of grains [5]. Specially, heat stress around flowering could reduce maize yield-related traits, including ear morphological traits, kernel weight, kernel numbers, and grain yield [6,7,8]. The reproductive growth stage, especially the V6 stage, is one of the key stages susceptible to heat stress. Therefore, high temperature in the V6 stage leads to the suppression of tassel development. Heat stress at the pre-anthesis stage is also detrimental to maize yield [9]. In addition, high temperature stress during the filling period inhibits the photosynthetic capacity of leaves, accelerates oxidative damage to cells, and causes premature leaf senescence [10]. Furthermore, the activities of enzymes involved in starch synthesis are suppressed and the starch average granule size and the proportion of long chains in amylopectin are increased by high temperature [11,12]. Moreover, heat shortens the active grain-filling period [13], ultimately leading to grain yield loss. Hence, it is crucial to have a better understanding of the molecular, biochemical, and physiological processes at different growth stages, especially during the reproductive and grain-filling stages, which ultimately impair the growth, development, and yield of maize under heat stress conditions.

Increasing research has indicated that high temperature stress affects the metabolic profile of maize. A series of metabolites related to heat stress have been detected, such as sugars, carbohydrates, amino acids, lipids, flavonoids, and organic acids. The strategy of combining transcriptome and metabolome analysis could better explain the transcriptional regulation of metabolic pathways [14]. By using this multi-omics strategy, numerous heat-stress-related genes have been identified [15,16].

Maize originated in tropical zones [8]. However, most maize cultivars are sensitive to heat, particularly during the pre-anthesis stage to the early grain-filling stage. During the maize breeding process, elite inbred lines were used to create hybrids, while others were discarded. Under long-term selection, this resulted in a constantly shrinking gene pool, and maize was faced with a major bottleneck of allelic abundance, making it susceptible to the impact of global warming. Different maize germplasms have different heat resistance abilities. Previous studies revealed a few maize germplasms exhibit better heat tolerance, such as the heat-tolerant lines PF5411-1 and DTPYC9F119 [17], and the maize heat resistance cultivars ZD309 and DKC7221 [18]. Heat stress affects the development of maize kernel numbers at the flowering and grain-filling stages, leading to yield reductions [6,8,19]. Enhancing maize tolerance to abiotic stress is a challenge to maize breeders.

In the Huang-Huai-Hai River Basin area of China, extreme high temperatures occur frequently in the maize-planting season. Heat tolerance is a crucial and complex quantitative trait for the growth of summer maize. Presently, the transcriptomic responses of maize to high temperature stress have been reported in a few studies. Most of them were focused on seedling-stage gene expression changes [20]. In this study, two commercial maize hybrids, ZD309 and XY335, were selected from four main planted maize hybrids in the Huang-Huai-Hai area of China based on high temperature resistance levels. A comparative transcriptomics and metabolomics analysis of ZD309 (heat-tolerant hybrid) and XY335 (heat-susceptible hybrid) between control and heat stress was carried out to elucidate the regulatory network in maize at the flowering stage. These results will be used to understand the molecular mechanism of maize heat tolerance and additionally provide valuable genes to improve targeted breeding.

## 2. Materials and Methods

### 2.1. Plant Materials, Growth Conditions and Heat Treatment

Commercial maize hybrids ZD958, ZD309 (ZD958 and ZD309 were supplied by Henan Academy of Agricultural Sciences, Zhengzhou, China), XY335, and DH605 (XY335 and DH605 were supplied by Shandong Denghai Seed Co., Ltd., Laizhou, China) were planted on farmland of Hebei Academy of Agriculture and Forestry Sciences (Shijiazhuang, Hebei, China, 38°07′ N, 114°22′ E). The farmland soil type was clay loam with 1.96% organic matter, 86.10 mg/kg available nitrogen, 32.75 mg/kg available phosphorus and 185.40 mg/kg available potassium at pH 7.74.

To estimate the critical period of heat stress effects, 160 plants of each hybrid were selected. The selected plants were divided into four groups under consistent growth conditions, with 40 plants each. Three groups were moved to a sunlight greenhouse for heat stress treatment at three different stages (pre-flowering stage, flowering stage and pre-grain filling stage). And other one group was placed in the field as the control. The daytime actual temperature in the sunlight greenhouse was 35–45.2 °C (outdoor air temperature 32.5–39.7 °C). And the nighttime actual temperature was 28–32.4 °C (outdoor air temperature 24.6–29.4 °C). The temperature in the greenhouse was controlled 3–5 °C higher than the field on average. All plants were adequately watered to prevent any water stress. The ears from the four groups were harvested at the kernel maturity stage for estimating the grain yield. The experiment was repeated three times.

For transcriptomics and metabolomics analysis, the hybrid ZD309 and XY335 were germinated and divided into two groups after selected under consistent growth conditions. The selected plants were placed in the field. At flowering period, the control group was continue placed in the field, and the other group was covered by a homemade facility for heat stress treatment. The homemade facility which made by resin film and steel frame structure with a transmittance of 95% to produce temperature and humidity control facilities, automatic heating devices are placed inside the facilities, and a 30% gap is reserved at the top of the shed for ventilation. At the same time, high-power ventilation fans are installed for air circulation. Continuous high temperature treatment for 7 days, with a day and night temperature controlled at 44/28 °C (day/night). During the treatment period, the average day and night temperature under natural conditions was 39/24 °C (day/night). After heat stress, remove the film and allow it to grow naturally. All plants were adequately watered to prevent any water stress. The ear leaves of maize were sampled after heat treatment, with three biological replicates, and stored at −80 °C for the following experiments.

### 2.2. Measurement of Heat Stress-Related Physiological Parameters

The physiological parameter of the content of proline, malondialdehyde (MDA), superoxide dismutase (SOD) activity, peroxidase (POD) activity, soluble protein content, soluble sugar content were determined with ear-leaf samples of ZD309 and XY335. The measurements were conducted using the corresponding testing kit from SuzhouComin Biotechnology Co., Ltd. (Suzhou, China. Available online: http://www.cominbio.com/a/shijihe/, accessed on 15 January 2022) by following the manufacturer’s instructions. These testing kits include PRO-1-Y, MDA-1-Y, SOD-1-W, POD-1-Y, KT-1-Y and BCAP-1-W.

### 2.3. RNA Extraction and Sequencing

Total RNA of the samples was isolated from maize leaves of two materials with the Trizol Reagent (Invitrogen Life Technologies, Carlsbad, CA, USA). NanoDrop sepectrophotometer (Thermo Scientific, Waltham, MA, USA) were used to determine the concentration, quality and integrity of RNA. The libraryfragments were purified using the AMPure XP system (Beckman Coulter, Beverly, CA, USA) to select cDNA fragments of the preferred 400–500 bp in length. After purified (AMPure XPsystem, Beckman Coulter, Beverly, CA, USA) and quantified (Agilent Bioanalyzer 2100 system, Santa Clara, CA, USA), the cDNA libraries were then sequenced on NovaSeq 6000 platform (Illumina) Shanghai Personal Biotechnology Cp. Ltd. (Shanghai, China).

### 2.4. Transcriptome Analysis

Adapter sequences and low-quality reads were removed from raw Sequencing data by using FASTP (0.22.0). The B73 maize reference genome (B73 RefGen_V4, available online: https://download.maizegdb.org/Zm-B73-REFERENCE-GRAMENE-4.0/, accessed on 10 June 2023) was used to aligned clean reads using the HISAT2 V2.1.0 [21]. When the whole transcriptome was done, HTSeq (v0.9.1) statistics were utilized to compare the Read Count values on each gene as the original expression of the gene, and then used FPKM to standardize the expression. The principal component analysis (PCA) was performed by the R package PCAtools v2.8.0 [22].

The differentially expressed genes (DEGs) were filtered by R package DESeq2 v1.20.0 [23], based on |log_2_ (fold change)| > 1 and statistical significance (*p*-adj value < 0.05). Common and unique DEGs analysis between different samples was conducted by visualizing results acquired from the R package VennDiagram [24]. A k-means cluster analysis was performed based on the R package factoextra [25]. Gene Ontology (GO) enrichment was performed by using R package ClusterProfiler (v4.6.0), calculate *p*-adj value by hypergeometric distribution method (the standard of significant enrichment is *p*-adj value < 0.05). ClusterProfiler (v4.6.0) software was used to carry out the enrichment analysis of the Kyoto Encyclopedia of Genes and Genomes (KEGG) pathway of differential genes, focusing on the significant enrichment pathway with *p*-adj value < 0.05.

### 2.5. Validation of Transcriptome Results Using Real-Time RT-PCR Analysis

The same samples were used to evaluate the gene expression levels by qRT-PCR. Nine genes from DEGs were selected randomly. Total RNA of the tested samples was reverse transcribed to first-strand cDNA with a cDNA Reverse Transcription Kit (PrimeScriptTM RT Master Mix, Takara, Tokyo, Japan). qRT-PCR was conducted with a ChamQ SYBR qPCR Master Mix kit (Vazyme, Nanjing, China) and a 7500 Fast Real-Time PCR System (Applied Biosystems, Foster, CA, USA). Relative transcript levels were calculated according to the 2^−ΔΔCT^ method. *ACT2* gene was used as an internal control for its consistent expression. Three biological and technical replications were performed.

### 2.6. Metabolite Identification and Metabolome Analysis

The samples are freeze-dried by vacuum freeze-dryer (Scientz-100F), and then was crushed using a mixer mill (MM 400, Retsch) with a zirconia bead for 1.5 min at 30 Hz. Briefly, 100 mg of lyophilized powder was dissolved with 1.2 mL 70% methanol solution, vortex 30 s every 30 min for 6 times in total, stored overnight at 4 °C. After centrifugation at 12,000 rpm for 10 min, the sample extracts were filtrated (SCAA-104, 0.22 μm pore size; ANPEL, Shanghai, China, http://www.anpel.com.cn/, accessed on 10 June 2023) and analyzed using an UPLC-ESI-MS/MS system (UPLC, SHIMADZU Nexera X2, www.shimadzu.com.cn/, accessed on 10 June 2023; MS, Applied Biosystems 4500 Q TRAP, www.appliedbiosystems.com.cn/, accessed on 10 June 2023) by an Agilent SB-C18 column (1.8 μm, 2.1 mm × 100 mm). LIT and triple quadrupole (QQQ) scans were acquired on a triple quadrupole-linear ion trap mass spectrometer (Q TRAP), AB4500 Q TRAP UPLC/MS/MS System, equipped with an ESI Turbo Ion-Spray interface, operating in positive and negative ion mode and controlled by Analyst 1.6.3 software (AB Sciex, Framingham, MA, USA).

Based on the Metware database (Metware Biotechnology Co., Ltd., Wuhan, China), the substance was characterized according to the secondary spectrum information, and the isotopic signal, the repeated signal containing K+ ions, Na+ ions, NH4+ ions, and the repeated signal of other fragments of larger molecular weight substances were removed during the analysis.

Metabolites from samples were used for hierarchical clustering analysis (HCA), principal component analysis (PCA), and orthogonal partial least squares discriminant analysis (OPLS-DA) using R software (v4.2.3) to study metabolite accession-specific accumulation. The metabolite data were log2- transformed (log_2_) and mean centering before OPLS-DA. Significantly regulated metabolites between groups were identified by variable influence on projection (VIP), and the threshold VIP ≥ 1 and absolute log_2_FC (fold change) ≥ 1 were applied. KEGG compound database (http://www.kegg.jp/kegg/compound/, accessed on 10 June 2023) and KEGG Pathway database (http://www.kegg.jp/kegg/pathway.html, accessed on 10 June 2023) with a *p*-adj value < 0.01 were used to identified and annotated metabolites and genes.

### 2.7. Statistical Analysis

All data obtained from yield, physiological parameters and qRT-PCR analyses were analyzed by Student’s t-test and one-way variance analysis (ANOVA) using software SPSS 19.0 (IBM, New York, NY, USA). *p*-value < 0.05 indicates the statistical differences to reach the significant different level, *p*-value < 0.01 for very significant different levels.

## 3. Results

### 3.1. Estimation of the Effects of Heat Stress on Different Stages of the Flowering Period

To estimate the most critical stage of heat stress effects on the flowering period, four commercial hybrid cultivars (ZD958, ZD309, XY335 and DH605) were selected for subjecting to heat stress at three time points: the pre-flowering (HS1), flowering (HS2) and pre-grain filling (HS3) stages. The heat-tolerant coefficient is represented as HS grain yield/CK grain yield (Table 1). The grain yield of four hybrid cultivars decreased under heat stress, with the most repressed occurring at flowering stage. Among the four tested varieties, XY335 was the most sensitive to heat stress, with a yield loss of 38.76%, 61.56% and 56.98% compared with the control at the three timepoints, while ZD309 exhibited a more stable yield, which indicates its heat tolerance under heat stress.

### 3.2. Evaluation of the Physiological Effects of Heat Stress at Flowering Stage

**Figure 1:**
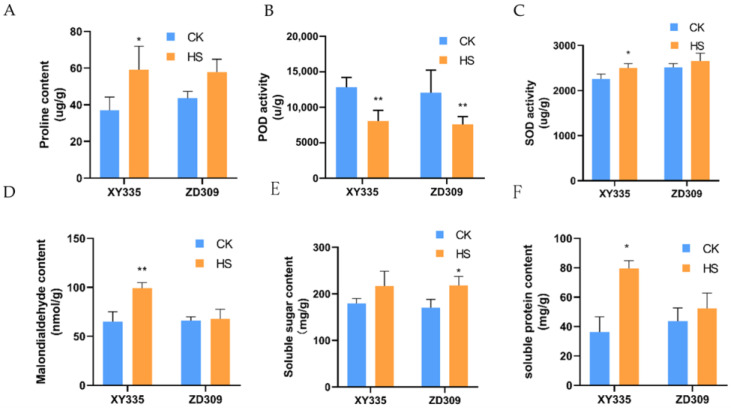
The effects of high temperature stress on the physical parameters of maize in XY335 and ZD309. (**A**) proline content, measured in ug/g, indicates the content in 1 g of sample’s fresh weight; (**B**,**C**) POD and SOD activity, measured in U/mg, indicates the respective activity units detected in 1 mg of sample’s fresh weight; (**D**) malondialdehyde content, measured in µmol/mg, indicates the respective content in 1 mg of sample’s fresh weight; (**E**,**F**) soluble sugar content and soluble protein content, measured in mg/g, indicates the respective content in 1 g of sample’s fresh weight; * and ** indicate that the corresponding physical character in heat-stressed maize plants exhibit significantly and very significantly different compared to the control at *p* < 0.05 and *p* < 0.01 levels, respectively.

Various physiological processes were activated under heat stress in maize. To verify the effect of heat stress on the flowering period of maize, samples were taken from the ear-leaf of maize and the physiological parameters were determined (Figure 1). Under heat stress treatment, the proline content in XY335 and ZD309 were both increased, and it was significantly increased in XY335. Malondialdehyde (MDA) content strongly increased in XY335, while it subtly increased in ZD309. The soluble protein contents, soluble sugar contents and SOD enzyme activities of XY335 and ZD309 increased under heat treatments. In contrast, the POD enzyme activity decreased significantly in both ZD309 and XY335. Overall, ZD309 shows superior heat tolerance and differences in physiological parameters compared to XY335.

### 3.3. RNA Sequencing Analysis for Two Hybrids at Flowering Stage under Heat Stress

To reveal the underlying mechanisms of heat tolerance in maize, two cultivars exhibiting diverse levels of resistance to heat, XY335 for heat susceptibility and ZD309 for heat resistance, were chosen for stressing under high temperature. Transcriptome of XY335 and ZD309, with and without heat stress, were performed on three biological replicates. From 12 samples, a total of 90.70 GB of raw data was collected, and 84.92 GB of clean data were produced after removing the low-quality data, with Q30 range from 93.57% to 94.53%. Clean reads were mapped to the reference genome, with between 95.97% and 96.22% found to be unique.

**Figure A1:**
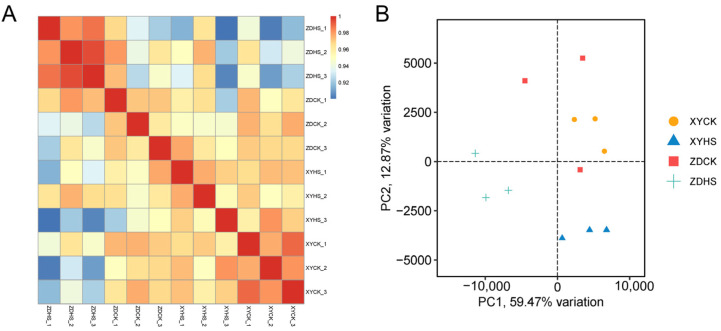
Spearman correlation coefficient (SCC) and Principal component analysis (PCA) of transcriptome analysis in XY335 and ZD309. (**A**) Spearman correlation coefficient (SCC) of gene expression profiles between samples. ZDHS refers to the heat treatment group of ZD309, ZDCK refers the control group of ZD309, XYHS refers to the heat treatment group of XY335, and XYCK refers the control group of XY335. are samples of two hybrids ZD309 (ZD) and XY335 (XY) with (HS) or without (CK) heat stress. The color of scale at the right of the figure indicates the coefficient value between each sample. (**B**) Principal component analysis (PCA) of samples distinguished by different colors with three biological repeats.

Reads were further mapped to the genome and a total of 39,591 genes were found in this study. Expressed genes were filtered using the total sum value of FPKM in all samples > 1 and 29,693 genes were obtained. Spearman correlation coefficient (SCC) and principal component analysis (PCA) were utilized to gain a global overview of the transcriptomic differences (Figure A1). Correlation values in samples of the same cultivar all exceeded 0.95, which indicates the good repeatability. PCA showed that separation of XY335 and ZD309 according to PC1 (59.47% variation), may be due to the variation between cultivars and treatments. In conclusion, differences were found between both treatment groups and cultivars.

### 3.4. Identification of DEGs at Flowering Stage for ZD309 and XY335

**Figure 2:**
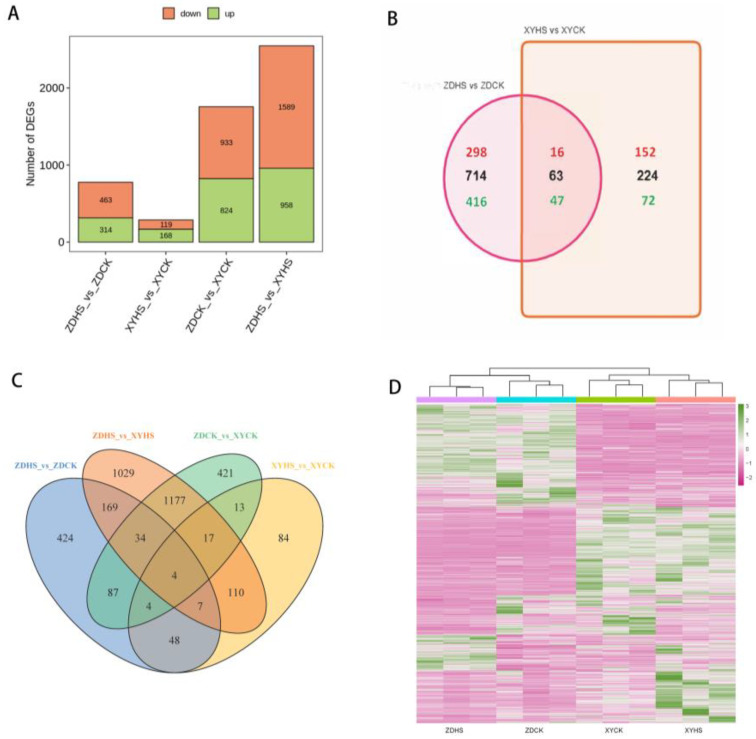
Transcriptome analysis for XY335 and ZD309 in response to heat stress. (**A**) Number of differentially expressed genes (DEGs) compared between different samples. (**B**,**C**) Venn diagrams showing the number of DEGs detected during analysis based on cultivar and heat treatment. Black numbers in parentheses represent totals, red and green numbers represent up- and down-regulated genes, respectively. (**D**) Hierarchical clustering of the up- and down-regulated genes. Purple and green lines in the heatmap represent the up- and down-regulated genes, respectively.

**Figure 3:**
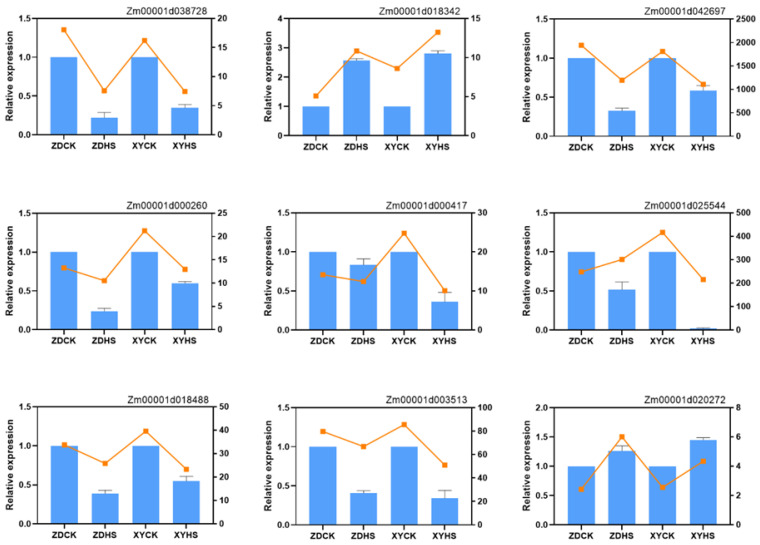
Verification of RNA-seq by qRT-PCR. Expression patterns of 9 randomly selected genes measured using the 2^−ΔΔCT^ method. The histograms in blue represented RT-PCR results with Error bars showing the means ± SEM. The orange lines represented the average values of FPKM for each sample.

To determine the genes with altered expression levels under heat stress for ZD309 and XY335, we studied DEGs at flowering stage between control and heat stress. A Venn diagram was used to identify DEGs with overlap under different conditions (Figure 2B,C). In ZD309, 777 unique genes were significantly responsive to heat stress including 314 up-regulated and 463 down-regulated DEGs, while 287 DEGs were detected in XY335 with 168 up-regulated and 119 down-regulated (Figure 2A). Among the differentially expressed genes that specifically respond to heat stress, 16 up-regulated and 47 down-regulated genes were identified as common between ZD309 and XY335. In ZD309, the expression of 714 genes changed significantly to heat stress but did not significantly respond in XY335. By contrast, there were 224 genes that responded to heat stress in XY335 but not in ZD309. Moreover, 1757 (824 up-regulated and 933 down-regulated) and 2547 (958 up-regulated and 1589 down-regulated) DEGs were identified in the pairwise comparison of ZDCK vs. XYCK and ZDHS vs. XYHS, respectively. Hierarchical clustering methods were used to provide an overview of the patterns of differentially regulated genes expression for each sample (Figure 2D). The expression patterns indicate the genes that may play a crucial role in heat responses and heat tolerance. The results reveal there are interspecific differences between ZD309 and XY335. The mechanisms for responding to heat may be partially identical between the two cultivars. To validate the reliability of the RNA-seq results, nine randomly selected DEGs were chosen and analyzed with qRT-PCR (Figure 3).

**Figure 4:**
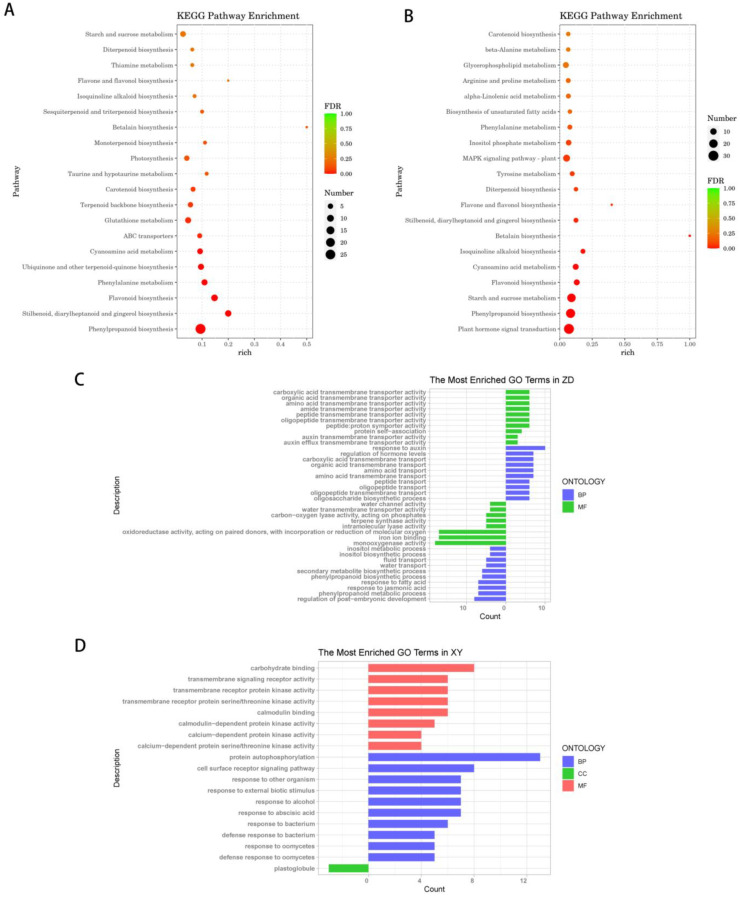
Significantly enriched terms in KEGG and GO analysis of DEGs in ZD309 and XY335. (**A**) KEGG enrichment in XY335. (**B**) KEGG enrichment in ZD309. (**C**,**D**) GO enrichment of ZD309 (ZD) and XY335 (XY). GO terms were subclassified into three categories distinguished by different colors: biological processes (BP), cellular components (CC), and molecular function (MF).

All DEGs were subjected to GO and KEGG enrichment analysis (Figure 4). For GO enrichment analysis, the up-regulated DEGs were found to be enriched in oligosaccharide biosynthetic process, response to auxin, amino acid transmembrane transport, response to hydrogen peroxide, and trehalose biosynthetic process terms in ZD309. In ZD309, the down-regulated DEGs were enriched in phenylpropanoid metabolic process, secondary metabolite biosynthetic process, response to jasmonic acid, response to fatty acid, monooxygenase activity, and oxidoreductase activity terms. In XY335, the up-regulated DEGs were enriched in protein autophosphorylation, response to abscisic acid, defense response, calmodulin-dependent protein kinase activity, and calmodulin binding. Meanwhile, down-regulated genes were enriched in plastoglobule, chloroplast stroma, and iron ion binding terms.

For KEGG pathway analysis, the DEGs in ZD309 were mainly found to be involved in starch and sucrose metabolism, flavonoid biosynthesis, plant hormone signal transduction, and MAPK signaling pathway. In XY335, the DEGs were mainly found to be involved in photosynthesis, starch and sucrose metabolism, flavonoid biosynthesis and phenylpropanoid biosynthesis. These findings demonstrated that heat stress has strong effects on signal transduction, photosynthesis, starch and sucrose metabolism and protein processing.

### 3.5. Expression Clusters by k-Means

**Figure 5:**
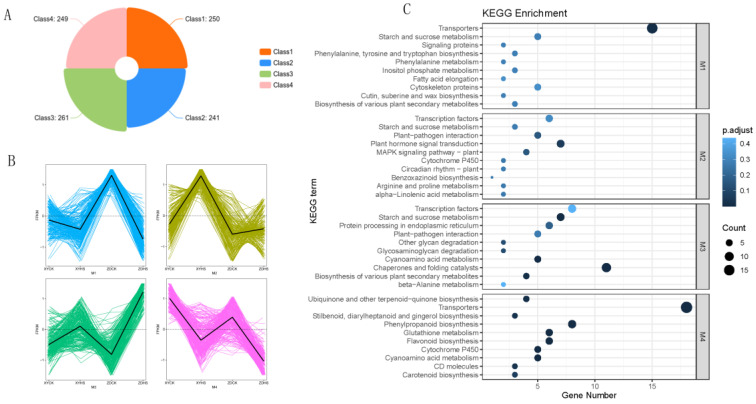
Cluster analysis of DEGs based on the k-means method. (**A**) Orange bars stand for DEGs expression pattern of both XY and ZD under control and heat stress. Cluster results of 1001 DEGs into four groups at different conditions. (**B**) Four clusters (M1–M4) based on the k-means algorithm. The *Y*-axis stands for scaled FPKM. Gene expression profiles in the line plots are shown in gray, and the mean values are shown in red for each cluster. (**C**) KEGG of four cluster of DEGs.

**Figure A2:**
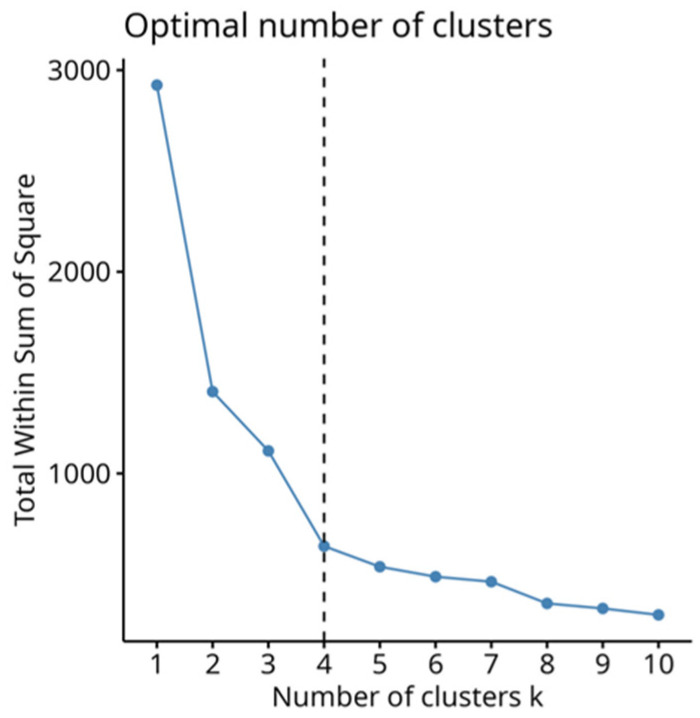
The optimal k value determined by the elbow method.

To study the genes expression patterns in ZD309 and XY335 under heat stress conditions, k-means clustering analysis was performed. All DEGs between the ZD309 and XY335 following heat stress treatment were further categorized into four clusters (Figure 5A,B). The optimal k value (4) was determined using the elbow method (Figure A2). Of the four clusters, M1 (250) and M4 (249) represent genes that were down-regulated under heat stress, whereas M2 (241) and M3 (261) represent genes that were up-regulated. In the up-regulated clusters, genes in M3 were specifically highly expressed in ZD309, and genes in M2 were specifically highly expressed in XY335.

We then conducted KEGG enrichment analyses for clusters based on gene expression patterns to differentiate genes linked with heat resistance and determine the top 10 enriched terms (Figure 5C). Cluster M2 and M3 represent genes that were up-regulated under heat stress. While genes in cluster M2 were greatly induced in cultivar XY rather than ZD, genes in cluster M3 increase more sharply in cultivar ZD. Cluster M3 consists of genes in ZD309 with the highest expression after heat treatment. Enrichment analysis showed that this cluster is enriched in terms of transcription factors, and starch and sucrose metabolism. Cluster M2 consists of genes with the highest expression in the XY335, in which the terms of transcription factors, starch and sucrose metabolism, plant hormone signal transduction, MAPK signaling pathway, and arginine and proline metabolism were enriched. These results indicate that starch and sucrose metabolism may play crucial roles in heat resistance, and that the divergence in expression levels of their related metabolism between the ZD309 and XY335 may explain the distinct levels of resistance.

### 3.6. Specific Responses of Heat Sensitive Maize to Heat Stress

**Figure 6:**
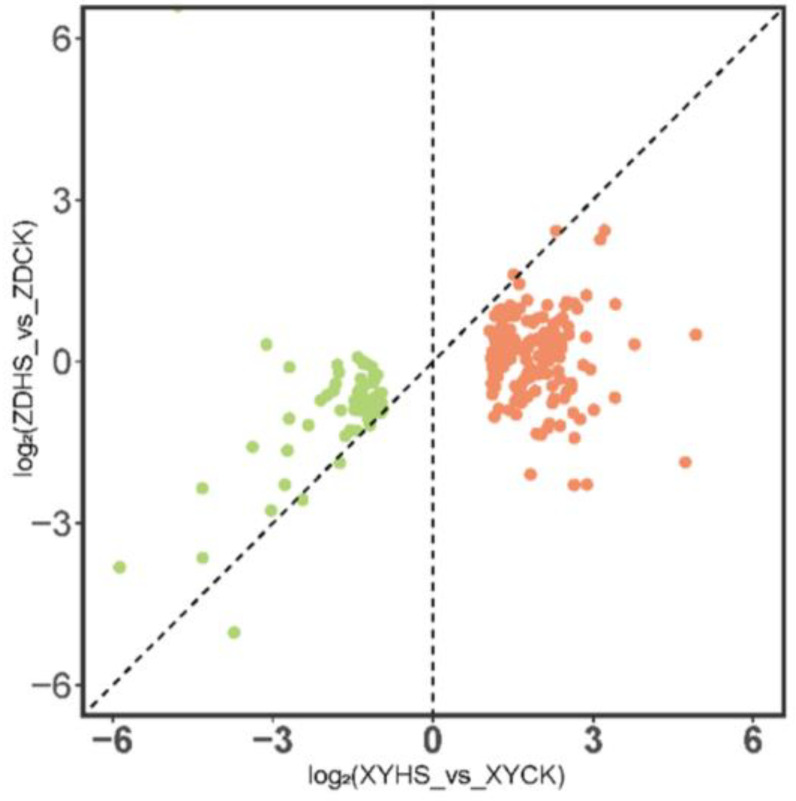
Scatterplot of the log_2_ fold changes in gene expression in response to heat stress in the XY335 vs. ZD309 for the 224 genes that were unique to XY335 heated vs. control comparison. The red and green spots represent the up- and down- regulated genes, respectively.

In this study, ZD309 and XY335 exhibited different transcriptome responses to high temperature stress. In XY335, 224 unique genes exhibited a notable response to high temperature stress, while they showed no significant pattern of differential expression in ZD309, due to the failure to meet the criteria of the absolute |log_2_ (fold change)| > 1 or statistical significance (*p*-adj value < 0.05). By analyzing the expression levels of these genes, it was observed that the total of 224 unique genes in XY335 exhibited higher (152 up-regulated) or lower (72 down-regulated) expression levels compared to ZD309 (Figure 6), and the lack of significant change in ZD was mainly caused by lower fold change of expression, not higher variance. We attempted to further understand the expression patterns of these 224 genes between ZD and XY under high temperature treatment (Figure 6).

**Figure 7:**
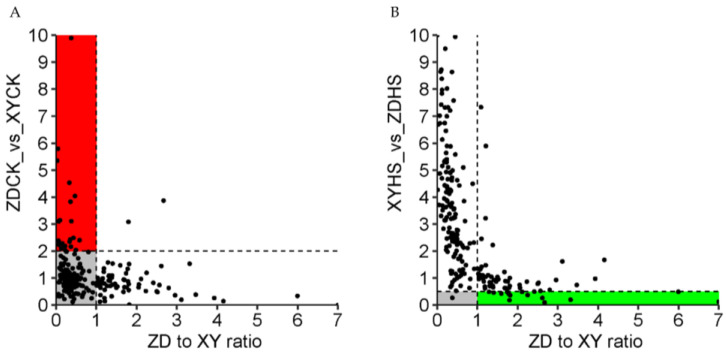
Scatterplot comparing the relative ratio of heat-to-control fold changes in expression between ZD and XY (on the x axis) to the XY to ZD control expression ratio (on the y axis) (**A**), and to the XY to ZD expression under heat stress (on the y axis) (**B**). The red and green portions of the graph represent the genes that are potentially frontloaded and stress indicators in expression, respectively.

Further, we compared the expression levels of these uniquely up-regulated genes in XY with ZD and XY under control conditions, simultaneously comparing the ratio of changes in these genes in ZDHS vs. ZDCK and XYHS vs. XYCK (Figure 7). The results show that 23 of the 152 up-regulated genes in XY exhibit higher expression levels (fold change ratio > 2) under heat stress (Table 2). However, before heat stress, these genes had higher expression levels in ZD than in XY, but no significant changes were found under heat stress. This relationship indicates that the expression levels of these genes were higher in resistant hybrid before being subjected to heat. Moreover, the resistant hybrid maintained a high expression level, but there was no significant change in these genes under heat stress. Conversely, in susceptible varieties, the expression levels of these genes were significantly up-regulated after stress, but maintained at a relatively low level before stress. These genes are potentially frontloaded in expression and may be related to heat resistance in maize.

We also compared the expression levels of those genes that were uniquely down-regulated in XY335 under high-temperature conditions in ZD309 and XY335, and jointly compared the change ratios of these genes in ZDHS vs. ZDCK and XYHS vs. XYCK. The results showed that a total of 15 genes displayed decreased expression levels after being subjected to high temperature stress in XY335 (fold change ratio > 2), while there was no significant expression change in ZD309 (Table 3). This relationship indicated that under high temperature stress, these genes were significantly down-regulated in sensitive varieties, while their expression levels remain unchanged in resistant varieties, which indicated that there was a correlation between these genes and heat tolerance in maize.

### 3.7. TFs Identified in the Response to Heat Stress

**Figure 8:**
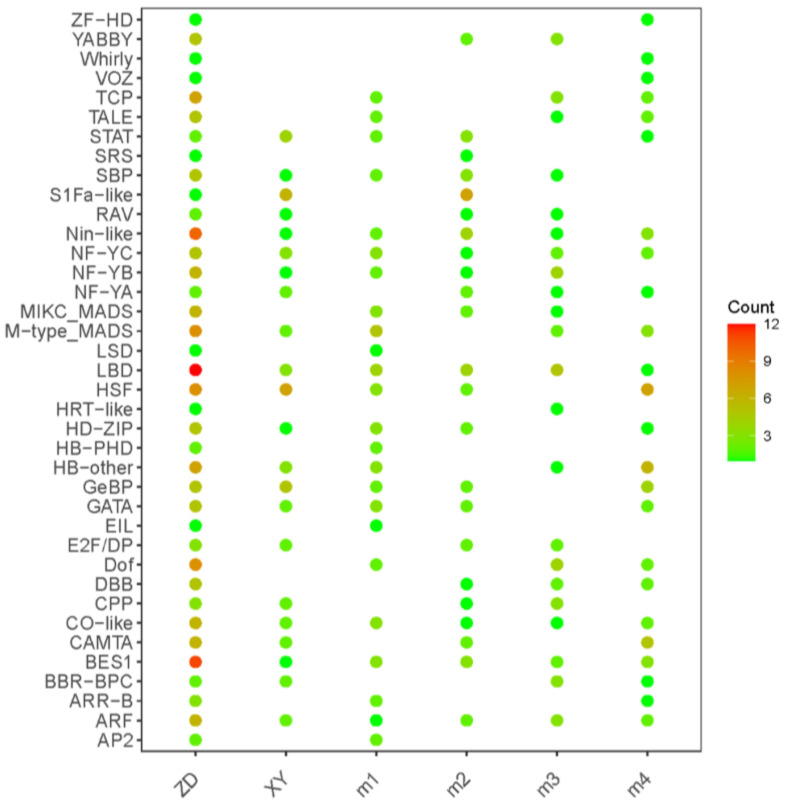
The identification of transcription factors (TFs). Distribution of TF families in the four clusters and two cultivars. The color represents the number of genes in each TF family.

To determine the transcription factors involved in heat stress response in each hybrid and cluster, all DEGs were compared to the iTAK database based on HMMER. Of the 437 TFs identified in the iTAK database, 170, 55, 58, 51, 47, and 56 predicted TFs from 38 distinct families were present in ZDHS vs. ZDCK, XYHS vs. XYCK, M1, M2, M3, and M4 (Figure 8). Heat stress transcription factors (HSFs) are major regulators of the plant heat stress response [26]. SQUAMOSA promoter-binding proteins (SBPs) form a major family of plant-specific transcription factors related to flower development [27]. The gaseous phytohormone ethylene regulates various processes related to the growth, development, and stress response of higher plants. A largely linear pathway of ethylene signaling has been revealed by a number of molecular genetic studies. In the pathway, ethylene-insensitive3 (EIN3) and EIN3-like (EIL) proteins are the key transcription factors. Some *HSP*, *SBP* and *EIL* transcription factors were identified according to the RNA-seq results, 15 *HSP* genes (8 in ZD309 and 7 in XY335), 6 *SBP* genes (5 in ZD309 and 1 in XY335), and 1 *EIL* gene (in ZD309) genes were obtained. These genes are potential candidates for the regulation of maize heat tolerance.

### 3.8. The Metabolic Analysis of ZD309 and XY335 in Response to Heat Stress

**Figure 9:**
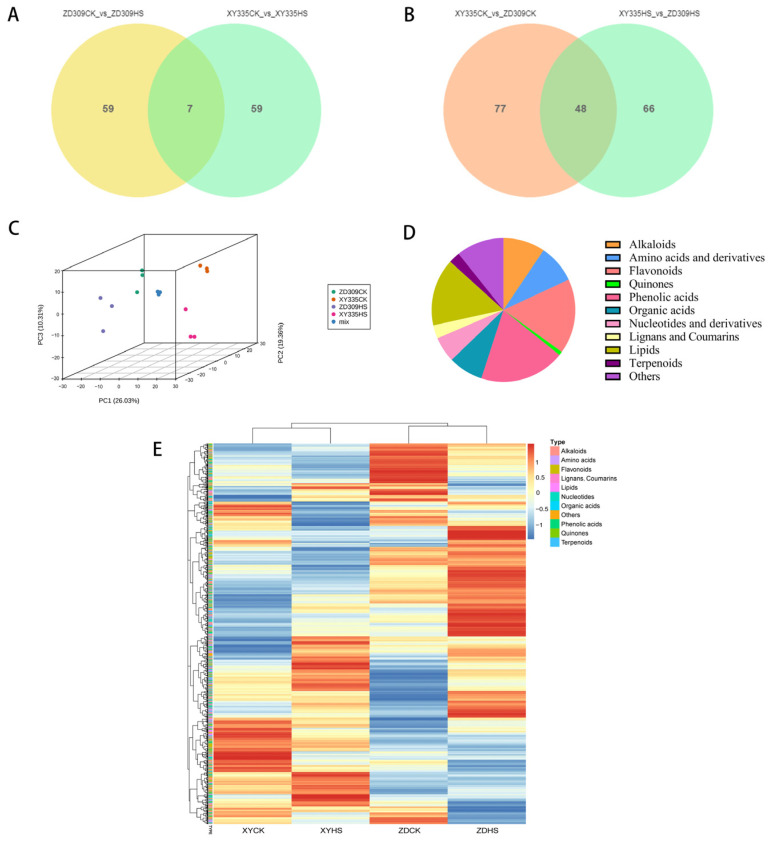
DEMs identifications within ZD309 and XY335. (**A**) Venn diagram showing the common and unique DEMs between ZD309 and XY335 under heat treatment. (**B**) Orthogonal Partial Least Squares-Discriminant Analysis (OPLS-DA) of metabolites in ZD309 and XY335 between control and heat stress. (**C**) principal component analysis of DEMs. PCA analysis showed a discrete trend among the four groups of metabolites, indicating that there were differences in their metabolites. (**D**) overview of annotated metabolites (**E**) Heat map of all identified metabolites in ZDCK, ZDHS, XYCK and XYHS. with or without heat treatment. ZDCK, ZDHS, XYCK and XYHS are samples of two hybrids ZD309 (ZD) and XY335 (XY) with (HS) or without (CK) heat stress.

To further investigate the metabolites of ZD309 and XY335 under heat stress, widely targeted metabolome analysis was performed using an LC–ESI–MS/MS system. A total of 1062 metabolites were identified in all samples. Principal component analysis (PCA) resulted in an obvious separation between XY335 and ZD309 (Figure 9C), with 27.68% for the first principal component (PC1) and 20.71% for the second principal component (PC2). The PCA score plots indicate that there is good repeatability within the group of samples used for metabolomics analysis demonstrate, as well as good dispersion between groups and sample specificity. A total of 1062 metabolites included in amino acids and derivatives (8.66%), phenolic acids (19.21%), nucleotides and derivatives (5.74%), flavonoids (16.85%), quinones (0.85%), lignans and coumarins (3.01%), others (10.73%), alkaloids (9.42%), terpenes (2.54%), organic acids (7.82%), and lipids (15.16%) were identified (Figure 9D,E). Other categories include vitamins (15.79%), sugars and alcohols (61.40%), and others (22.81%). The heatmaps of all metabolites were used to display changes in the metabolites of ZD309 and XY335 under HS. The results clearly indicate a notable disparity in metabolite changes between the two hybrids, indicating there is a significant difference in their responses to heat.

**Figure 10:**
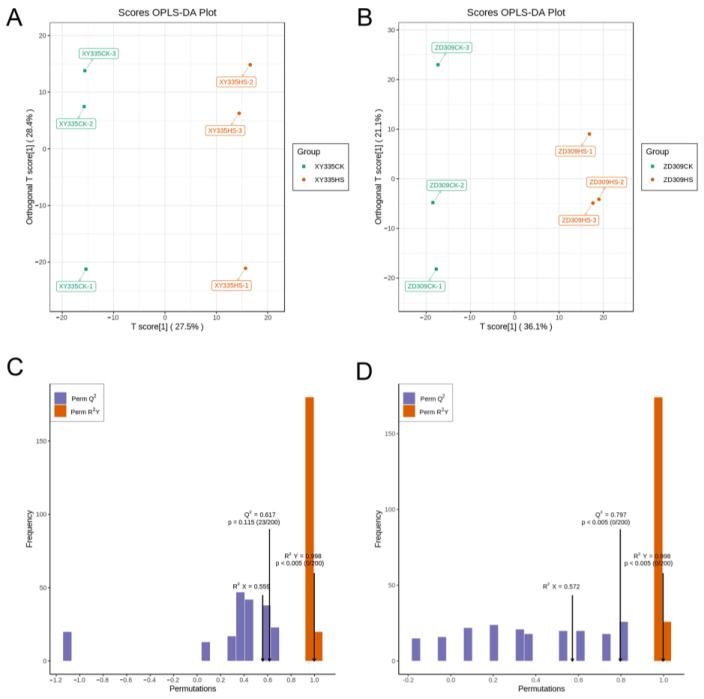
OPLS-DA (Orthogonal Partial Least Squares-Discriminant Analysis) of metabolites in ZD309 and XY335 between control and heat stress. (**A**,**B**) OPLS-DA score for XY335 and ZD309 (**C**,**D**) OPLS-DA Permutation Test of XY335 and ZD309.

Orthogonal partial least squares-discriminant analysis (OPLS-DA) was implemented to analyze the differences between samples (Figure 10). R^2^X, R^2^Y, and Q^2^ were 0.559 and 0.572, 0.998 and 0.998, 0.617 and 0.797 in the pairwise comparison of XYCK vs. XYHS and ZDCK vs. ZDHS, respectively. The OPLS-DA permutation test results indicate that the data were well matched with OPLS-DA model. Based on a log_2_FC (fold change) ≥ 1 and VIP value ≥ 1, the metabolomics results show that there were 66 differentially expressed metabolites (DEMs) in XY335 after high-temperature stress, of which 12 were down-regulated and 54 were up-regulated; meanwhile there were 66 DEMs, of which 17 were down-regulated and 49 were up-regulated in ZD309. Of these DEMs, 7 detected DEMs (5 up-regulated and 2 down-regulated) were common to both XY335 and ZD309 under control and stress (Figure 9A). Furthermore, 125 (75 up-regulated and 50 down-regulated) and 114 (65 up-regulated and 49 down-regulated) DEGs were identified in the pairwise comparison of XYCK vs. ZDCK and XYHS vs. ZDHS, respectively (Figure 9B).

**Figure 11:**
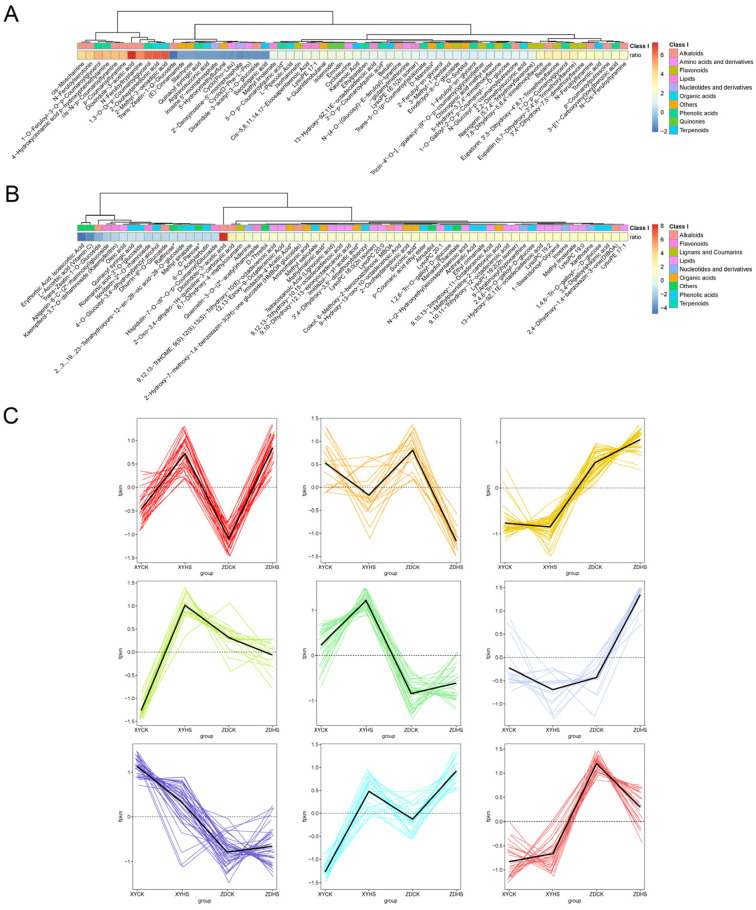
Metabolomic profiles of maize ear-leaves under heat stress. Select conditions were VIP ≥ 1 and log_2_FC1 ≥ 1. (**A**,**B**) Differentially expressed metabolites in ZD309 and XY335 using Z-score standardization. (**C**) Cluster analysis of 262 differential metabolites, which were divided into 9 groups.

In order to study the relative changes in metabolites content in the different groups, a total of 262 significant different metabolites were clustered into nine groups based on k-means clustering analysis. The relative contents of all differential metabolites were normalized using Z-score (Figure 11). Of the nine clusters, C1, C5 and C8 represent metabolites that were up-regulated with heat stress in both hybrids. In the up-regulated clusters, a total of 31 metabolites in C1 were specifically highly increased in both XY335 and ZD309 after heat stress, including lipids (41.93%), phenolic acids (19.35%), nucleotides and derivatives (9.68%), organic acids (9.68%), alkaloids (6.45%), flavonoids (3.23%), coumarins (3.23%) and others (6.45%). Of these, Lmhp009464 (lysophosphatidylethanolamine, lyso-PE) was up-regulated in both ZD309 and XY335 in response to heat. In C8, mws0214 (D-sorbitol), mws1155(D-mannitol), pme2134 (D-threitol) exhibited higher concentrations in the stress treatment compared to the control. C2 was the only cluster in which the metabolites were down-regulated in both hybrids. Additionally, mws1589 (D-panose) and pme2125 (raffinose), both clustered in C2 and were negatively affected by heat in ZD309.

### 3.9. Combined Transcriptomic and Metabolomic Analysis after Exposure to Heat Stress

Correlation analysis between two omics was performed to identify the highly associated DEGs and metabolites. Interaction networks may be built to help us better understand the underlying mechanisms of heat stress and functional relationships. The correlation values between a total of 1001 DEGs identified in two cultivars and all of the 1062 identified metabolites were measured, and a total of 40 DEGs and 41 metabolites were considered to be highly associated (cor^2^ > 0.9, *p* < 0.05). Of the 84 correlations, 15 were negative. Further analysis showed there were 10 flavonoids and 3 saccharides among the 40 strong DEG-related metabolites, which may indicate the potential roles of pathways related to flavonoid and saccharide in heat resistance.

**Figure 12:**
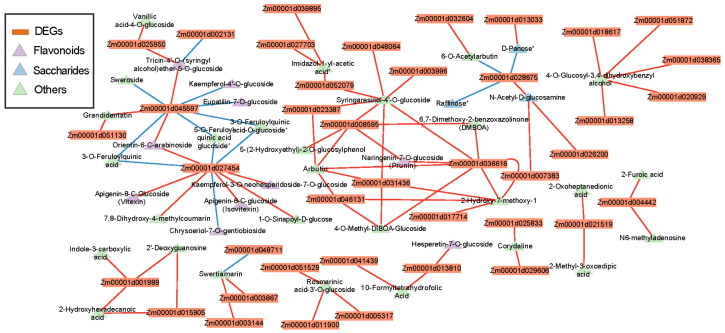
Correlation network of DEGs and their highly related(cor^2^ > 0.9, *p* < 0.05) metabolites. Triangle represents pivotal metabolites in the networks, while orthogon indicates supporting genes on the edge. Blue lines represent down-regulated metabolites and red lines indicate up-regulated metabolites and genes.

**Figure A3:**
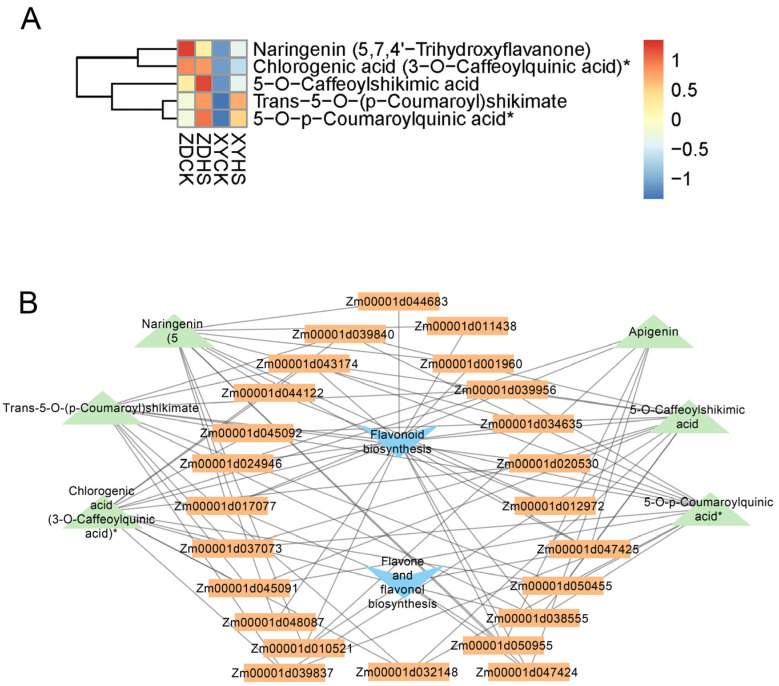
Analysis of interactions of differential expression genes (DEGs) and metabolites involved in flavonoids metabolism under heat stress. (**A**) The content of metabolites which identified in flavonoids metabolism using Z-score standardization. * represent significant differential expression. (**B**) Analysis of interactions between DEGs and metabolites involved in flavonoids metabolism.

To better understand the mechanism for response to heat in maize, KEGG markup language (KGML) was used for integrated analysis, which is a database containing information about the correlation between genes and metabolites in multiple pathways (Figure 12). Based on the results of KEGG enrichment analysis and gene-metabolite correlation analysis, we further focused on flavonoid-related biosynthesis. The interactions between genes and metabolites in phenylpropanoid/flavonoids metabolic pathways (zma00940, zma00941 and zma00944) were filtered according to the KEGG pathway XMLs (Figure A3). Then, all of the genes and metabolites identified in this study were mapped. The highest content for most of the metabolites involved in this network was for ZDHS. In contrast, the lowest content was found for XYCK. This may explain the high resistance of ZD309 to heat, based on its increase in flavonoids metabolites when confronting heat stress and, conversely, the sensitivity of XY335 based on its low background content.

**Figure A4:**
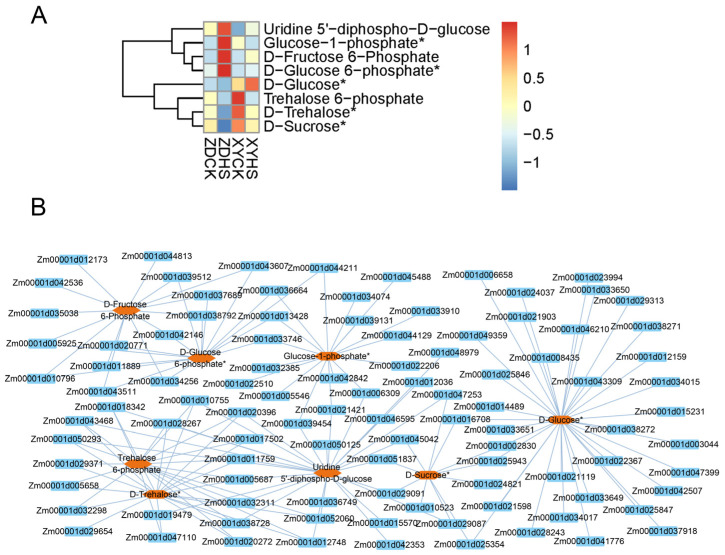
Analysis of interactions of differential expression genes (DEGs) and metabolites involved in starch and sucrose metabolism under heat stress. (**A**) The content of metabolites which identified in starch and sucrose metabolism using Z-score standardization. * represent significant differential expression. (**B**) Analysis of interactions between DEGs and metabolites involved in starch and sucrose metabolism.

Starch and sucrose metabolism pathways (zma00500) were also identified following transcriptome and KEGG enrichment analyses, and are considered to be some of the key pathways in heat stress. A network was built in this study and six identified metabolites were found (Figure A4). We found that the contents of four metabolites, uridine 5′-diphospho-D-glucose, glucose-1-phosphate*, D-fructose 6-Phosphate and D-glucose 6-phosphate*, reached the peak in ZDHS. Interestingly, the contents of three other metabolites, trehalose 6-phosphate, D-trehalose* and D-sucrose* were highest in XYCK. This may indicate the diverse functions of these saccharides.

**Figure A5:**
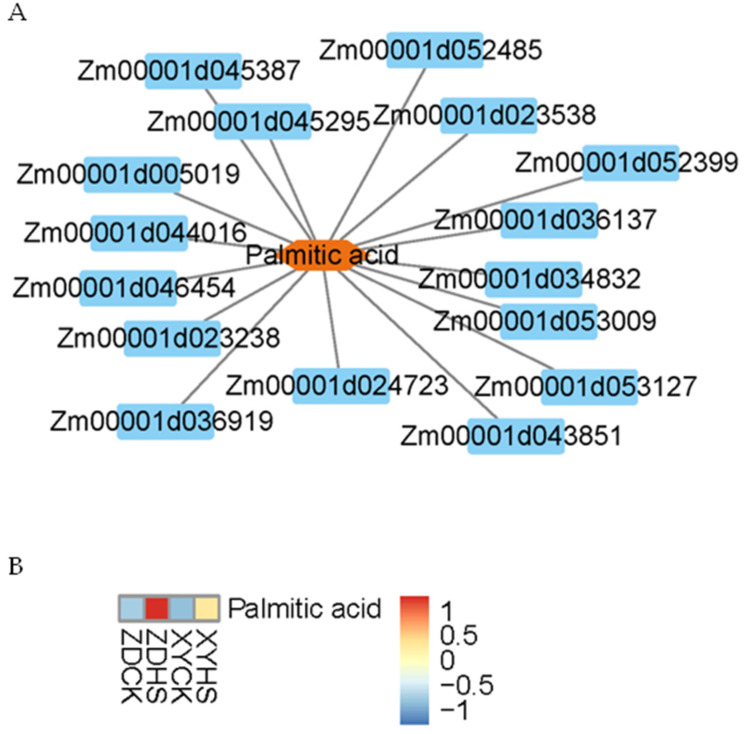
Analysis of interactions of differential expression genes (DEGs) and metabolites involved in lipid metabolism under heat stress. (**A**) The content of metabolites which identified in lipid metabolism using Z-score standardization. (**B**) Analysis of interactions between DEGs and metabolites involved in lipid metabolism.

Several studies have demonstrated that lipids are relevant to heat stress [28]. The network of interactions under lipid metabolism (zma00061, zma00062) was built as mentioned above (Figure A5). Unfortunately, one metabolite mapped to this, for most of the lipids in this network were not detected. Palmitic acid was increased under both XY335 and ZD309 cultivars, and ZD309 has the highest content. Considering its key function, palmitic acid may be crucial for plants to defend heat stress.

## 4. Discussion

With global warming, high temperatures have become one of the main factors affecting crop production. Heat stress has led to a significant loss in global crop yield. It also affects various physiological process in plants [18]. Several morphological, physiological, biochemical, molecular and yield-related traits and processes in maize are negatively impacted under heat stress conditions. Under high-temperature stress, the chlorophyll content and photosynthetic rate of maize are decreased, and the cell membrane and antioxidant system are damaged [4,17,29]. These physiological changes can affect the growth and development of maize, ultimately leading to a decline in yield [30,31,32]. Both short and long durations of high temperature lead to impairment of maize reproductive processes [5]. The sensitivity to high temperature also varies distinctly for each of the different growth stages. The flowering stage is one of the critical phases in maize that account for most of the yield losses due to heat stress [6]. Pollen formation, pollen development, and tassel development are extremely susceptible to heat stress [14,20,33]. Different genotypes of maize exhibit differences in heat tolerance. Comparing the response patterns between heat-resistant maize and heat-sensitive maize to high temperature will help in elucidating the molecular mechanisms of maize heat tolerance. In the present study, ZD309 and XY335 were selected from the main cultivated hybrids in the Huang-Huai-Hai area of China, representing resistance and sensitivity to high temperature during the flowering period, respectively. Studying the response mechanism of the main cultivated varieties to high temperature stress during critical growth stages will be helpful for maintaining maize production and reducing the risk of heat stress under a changing climate.

At the molecular level, maize reduces heat stress damage by regulating a series of specific genes and proteins. Exploring key heat stress response genes is beneficial for creating a heat-resistant maize germplasm. So far, researchers have validated some genes that could improve plant heat tolerance, including *ZmCDPK7* [34], *ZmDHAR* and *ZmADCS* [35], *ZmHUG1* [36], and *ZmHsf01* [37]. Moreover, GWAS and QTL were also used to identify heat-tolerant gene loci [38,39,40,41]. TFs can ensure that target genes are expressed at a specific intensity within a specific time and space [13,42]. A series of TFs are related to heat shock, such as HSF. In this study, a total of 38 TF families were identified, including the HSF and SBP TF families. Of these, the largest is the HSF family. HSF is significantly correlated with plant stress resistance [43]. Overexpression of *ZmHsf05* in Arabidopsis could increase the plant’s basal and heat tolerance [44]. Further analysis could be conducted on the differentially regulated HSF transcription factors, the results of which may contribute to improving the high-temperature tolerance of maize.

In heat-tolerant maize, the reduced heat stress reaction may be caused by two potential gene regulatory phenomena. Some of the genes could have a decreased response due to their higher constitutive expression levels in heat-tolerant maize under control conditions. These frontloaded genes may confer resilience by reacting faster, at the protein level, during instantaneous heat stress, thereby preparing individuals for frequently encountered stressful situations [45]. Therefore, frontloading may prepare individuals for the pressure they often encounter. Alternatively, some genes may maintain a certain level of expression after stress to reduce the damage of high temperature. In our experiment, ZD309, which has stronger heat resistance, may have experienced lower levels of physiological stress, so the expression changes in these “stress indicator” genes may be relatively small [46]. It is plausible that the more heat-tolerant hybrid experienced lower levels of physiological stress during our experiment, resulting in smaller changes in expression levels of these “stress-indicator” genes. These frontloaded and stress-indicator genes represent a valuable potential area for investigating mechanisms that differentiate reactions between stress-tolerant and stress-sensitive maize.

Under heat stress, plant response is a complex process at both cellular level and whole plant level. the transcriptome analysis showed many biological pathways were involved in ZD and XY cultivars under heat stress such as plant hormone signal transduction, flavonoid biosynthesis and starch and sucrose metabolism pathways while the diverse expression levels of genes in these pathways may be accountable for the different heat resistance under two cultivars. For instance, we found plant hormone signal transduction and starch and sucrose metabolism pathways were significantly enriched peculiarly in ZD309. These results indicated clues to explore the molecular basis of the differences in response to heat stress.

Combined Transcriptomic and Metabolomic Analysis has been proved to be an effective and powerful way to provided new information for mechanism research. Through Correlation analysis we found flavonoids and saccharides metabolites were highly correlated to the DEGs (Figure 12). We further noticed that the four flavonoids and four saccharides reached their peak contents in ZD under heat stress. Similar to our results, related transcriptome analyses have shown that saccharides metabolites and flavonoids play the crucial role in response to heat stress [47,48,49].

Lysophosphatidylethanolamine (lyso-PE) can enhance plants adaptation to abiotic stress, and it has been demonstrated that lyso-PE can enhance the tolerance of plants to heat stress [28]. In our study, we found that the content of lyso-PE in maize leaves of both ZD309 and XY335 was significantly increased after heat stress. Therefore, we inferred that lyso-PE is related to the heat resistance of maize. Sacchaides and flavonoids were identified in metabolomics analysis(Figure A3 and Figure A4), and both can enhance the tolerance of plants to temperature stress [50]. In our study, we found that the contents of uridine 5′-diphospho-D-glucose, glucose-1-phosphate*, D-fructose 6-phosphate, D-glucose 6-phosphate*, trehalose 6-phosphate, D-trehalose* and D-sucrose* in maize were significantly changed after heat stress (Figure A4). Therefore, we inferred that the response of these metabolites to heat stress may be useful in the heat tolerance of maize. Hence, genes involved in the galactose synthesis pathway could be utilized to improve the heat tolerance of maize in breeding.

## 5. Conclusions

High-temperature stress during flowering can impact the development of maize pollen and filaments, leading to a decrease in grain quantity and yield, ultimately resulting in yield loss. In this study, we uncovered the complex response mechanism to heat during maize flowering through transcriptomics and metabolomics analyses. Through transcriptomic data analysis, we identified many key genes involved in regulating various metabolic pathways related to heat tolerance response mechanisms in ZD309 and XY335. These genes could mediate the antioxidant reduction system, osmotic adjustment processes, plant hormone regulation, and photosynthesis-related pathways, which may play a crucial role in maintaining maize cell homeostasis and physiological processes under heat stress. In addition, metabolomic analysis revealed different metabolic characteristic in ZD309 and XY335 under normal conditions and heat stress. Products related to lipids, flavonoids, and energy metabolism underwent significant changes during the thermal response processes of these two experimental cultivars. These findings contribute to further understanding of the different responses of individual genotypes of maize to heat stress. Our results also provide valuable candidate genes for maize heat tolerance breeding.

## Figures and Tables

**Table 1:** Identification of heat stress critical stage on yield in 4 commercial hybrids at 3 different stages.

	Grain Yield (kg/ha)				Heat-Tolerant Coefficient (%)		
Varieties	CK	HS1	HS2	HS3	HS1	HS2	HS3
XY335	7318.2	4481.6	2813.5	3148.0	61.24	38.44	43.02
ZD958	6971.0	5785.9	4702.5	5928.0	83.00	67.46	85.04
ZD309	6875.0	6409.5	5974.4	6141.0	93.23	86.90	89.32
DH605	7182.0	5724.1	3363.0	6289.5	79.70	46.83	87.57

**Table 2:** A total of 23 potentially frontloaded genes screen from unique up-regulated genes in XY which exhibited higher expression levels (fold change ratio > 2) under heat stress.

Gene ID	Description
Zm00001d003110	Calmodulin-binding receptor-like cytoplasmic kinase 3
Zm00001d005788	Zinc finger protein 2
Zm00001d005823	Flavonoid 3-monooxygenase
Zm00001d008588	cytochrome P450 family 77 subfamily A polypeptide 5 pseudogene
Zm00001d016899	-
Zm00001d020277	Carbohydrate transporter/sugar porter/transporter
Zm00001d021879	NEP-interacting protein 1
Zm00001d021995	LOB domain-containing protein 38
Zm00001d023311	Os01g0112866 protein
Zm00001d026222	-
Zm00001d031726	Pyridoxal phosphate (PLP)-dependent transferases superfamily protein
Zm00001d036122	Glycosyltransferase family 61 protein
Zm00001d037680	Vegetative storage protein 2
Zm00001d043199	Disease resistance protein RGA4
Zm00001d043298	*p*-loop containing nucleoside triphosphate hydrolases superfamily protein
Zm00001d044010	Probable WRKY transcription factor 30
Zm00001d044826	NOD26-like membrane intrinsic protein2
Zm00001d047523	Probable WRKY transcription factor 30
Zm00001d047883	Probable carboxylesterase 17
Zm00001d048690	Serine carboxypeptidase-like 50
Zm00001d048702	benzoxazinone synthesis3
Zm00001d051194	Arginine decarboxylase
Zm00001d052518	Pollen Ole e 1 allergen and extensin family protein

**Table 3:** A total of 15 potentially stress indicator genes screen from unique down-regulated genes in XY which displayed decreased expression levels e (fold change ratio > 2) under heat stress.

Gene ID	Description
Zm00001d002131	-
Zm00001d008548	Bowman-Birk type wound-induced proteinase inhibitor WIP1
Zm00001d008764	UDP-glycosyltransferase 75B1
Zm00001d011063	Metallothionein-like protein 2C
Zm00001d016014	Type III polyketide synthase B
Zm00001d016016	Dynein light chain LC6%2C flagellar outer arm
Zm00001d017249	ammonium transporter2
Zm00001d020610	-
Zm00001d020623	Copper transporter 5
Zm00001d028815	Pathogenesis-related protein 10
Zm00001d036986	ABC transporter G family member 29
Zm00001d038750	ALA-interacting subunit 1
Zm00001d039643	UDP-glycosyltransferase 73D1
Zm00001d045991	-
Zm00001d048449	Senescence-associated protein

## Data Availability

The datasets supporting the conclusions of this article are included within the article (and its additional files). Sequencing database for maize could download from NCBI under the accession number PRJNA1047035, and the data will be shared on reasonable request of the corresponding author.
